# Germline Gene Editing in Chickens by Efficient CRISPR-Mediated Homologous Recombination in Primordial Germ Cells

**DOI:** 10.1371/journal.pone.0154303

**Published:** 2016-04-21

**Authors:** Lazar Dimitrov, Darlene Pedersen, Kathryn H. Ching, Henry Yi, Ellen J. Collarini, Shelley Izquierdo, Marie-Cecile van de Lavoir, Philip A. Leighton

**Affiliations:** Crystal Bioscience, Inc., Emeryville, California, United States of America; University Tuebingen, GERMANY

## Abstract

The CRISPR/Cas9 system has been applied in a large number of animal and plant species for genome editing. In chickens, CRISPR has been used to knockout genes in somatic tissues, but no CRISPR-mediated germline modification has yet been reported. Here we use CRISPR to target the chicken immunoglobulin heavy chain locus in primordial germ cells (PGCs) to produce transgenic progeny. Guide RNAs were co-transfected with a donor vector for homology-directed repair of the double-strand break, and clonal populations were selected. All of the resulting drug-resistant clones contained the correct targeting event. The targeted cells gave rise to healthy progeny containing the CRISPR-targeted locus. The results show that gene-edited chickens can be obtained by modifying PGCs *in vitro* with the CRISPR/Cas9 system, opening up many potential applications for efficient genetic modification in birds.

## Introduction

The chicken embryo is a key model system in developmental biology [1]. Fundamental contributions have been made in the areas of immunology, neurobiology, pattern formation, and others, taking advantage of the ease of access to the embryo in the egg. In human health, chicken eggs are the method of choice in the production of influenza vaccine. Despite the importance of chickens in research, the ability to produce birds carrying targeted genetic modifications such as knockouts and knock-ins for functional studies in whole animals has only recently become available [2]. Genome modification in chickens has been accomplished using germline stem cells, such as primordial germ cells (PGCs) or gonocytes, as intermediates [3–7]. Cultured PGCs can be transfected and injected into embryos, where they enter the germline, to obtain gene knockouts with standard homologous recombination vectors [8]. Targeting efficiencies of ~30% can be expected when using homology regions of about 7-8kb. Although the targeting efficiency is high, obtaining these long, isogenic homology regions can be labor-intensive. To make a targeted change to a gene, alternative approaches are now available, such as gene editing with site-specific endonucleases [4]. Gene editing is more accessible and quicker to implement than standard homologous recombination, especially for the clustered regularly interspaced short palindromic repeat (CRISPR) and CRISPR-associated nuclease (Cas9) system, which requires only a 20bp sequence to program the system.

CRISPR/Cas9 has been vigorously pursued as an efficient method for genetic modification in a wide variety of animals, including livestock species [9]. Cas9 nuclease creates double-strand breaks in DNA at a site specified by a 20nt guide RNA (gRNA). Resolving the double-strand break can be accomplished in two different ways: error-prone repair by non-homologous end joining (NHEJ), leading to small insertions/deletions, or by homology-directed repair (HDR) when a donor DNA is supplied along with the CRISPR/Cas9 [10,11]. In many animals to date, the CRISPR/Cas9 components have been directly injected into the zygote where they target the genome and result in animals carrying mutations on one or both sister chromatids [9,12]. To gain more control over the process, targeting in somatic cells such as fibroblasts can be used to produce either NHEJ or HDR-based mutations, followed by somatic cell nuclear transfer (SCNT) to produce live progeny carrying the mutations [13–15].

In the chicken, CRISPR/Cas9 has been used to knock out genes in somatic cells of the developing embryo [16], but no germline modifications have been reported. In birds, direct injection of constructs into the zygote and SCNT approaches are impractical, owing to the structure of the zygote, the opacity of the oocyte, and the difficulty in retrieving, manipulating, and culturing early embryos to term [17,18]. Direct *in vivo* transfection of germline cells in embryos by lipofection is an alternative strategy to introduce constructs but the efficiency of transfection is low [19,20]. Here, we show that CRISPR/Cas9 can be used in chicken PGCs and is an efficient route to editing the genome of transgenic birds. This expands the range of technologies that can be used to introduce genetic modifications in PGCs, and will enable further functional genomic studies using the chicken.

## Results

**Fig 1:**
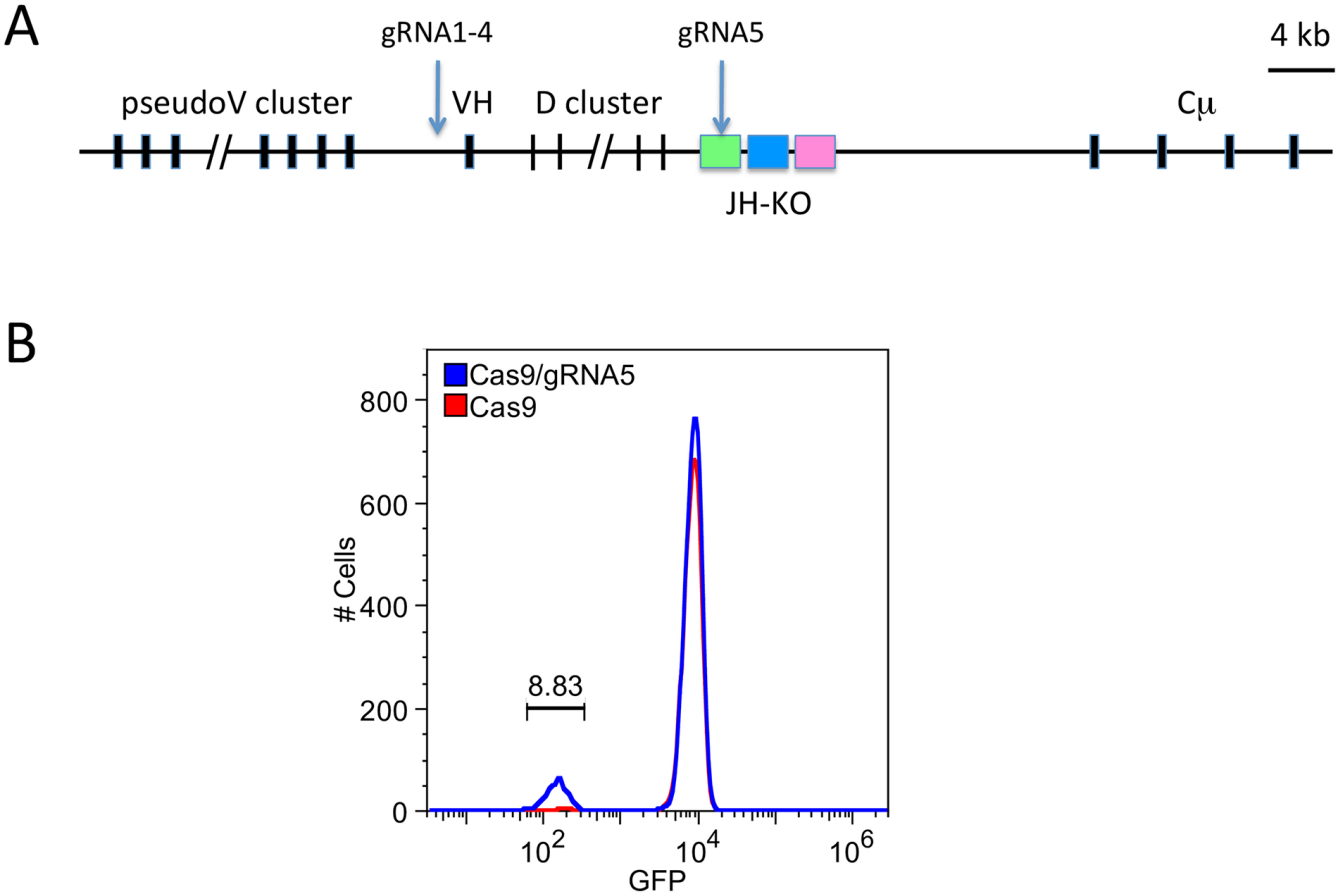
Strategy for CRISPR targeting. A. Diagram of the chicken IgH locus present in PGC line 472–138 used for CRISPR targeting. The IgH locus contained a previously obtained knockout of the JH gene segment (JH-KO), between the D cluster and the constant regions (only Cμ is shown), which was replaced with a selectable marker cassette. gRNAs 1 through 4 were designed to target a region upstream of the single functional VH region (indicated with an arrow), and gRNA5 was designed to target the EGFP gene. B. PGCs of line 472–138 were transiently transfected with a construct containing Cas9 or Cas9/gRNA5, specific for EGFP. After 9 days in culture, the cells were analyzed by flow cytometry for loss of green fluorescence.

To test whether CRISPR/Cas9 could be used to edit the PGC genome, we first performed an experiment to inactivate an enhanced green fluorescent protein (EGFP) transgene inserted in the IgH locus. The EGFP gene is part of a selectable marker cassette that was used to knock out the JH gene segment in the JH-KO PGC cell line 472–138 (Fig 1A). A previously described gRNA (gRNA5)[21] to EGFP was cloned in a U6-expression vector (GE6) that also carries the wild type Cas9 nuclease. PGCs were transiently transfected with constructs encoding Cas9 with and without gRNA5. Nine days after transfection, the cell population was analyzed by flow cytometry, and ~9% of the cells in the Cas9/gRNA5 population had lost EGFP expression compared to the control transfection (Fig 1B).

To produce genetically-modified birds with CRISPR/Cas9, the modification produced in PGCs must be passed through the germline to the next generation. For this purpose, clonal populations in which every cell carries the desired mutation are preferred. We designed a drug selection strategy in combination with CRISPR/Cas9 to select and grow clones carrying the modification.

**Fig 2:**
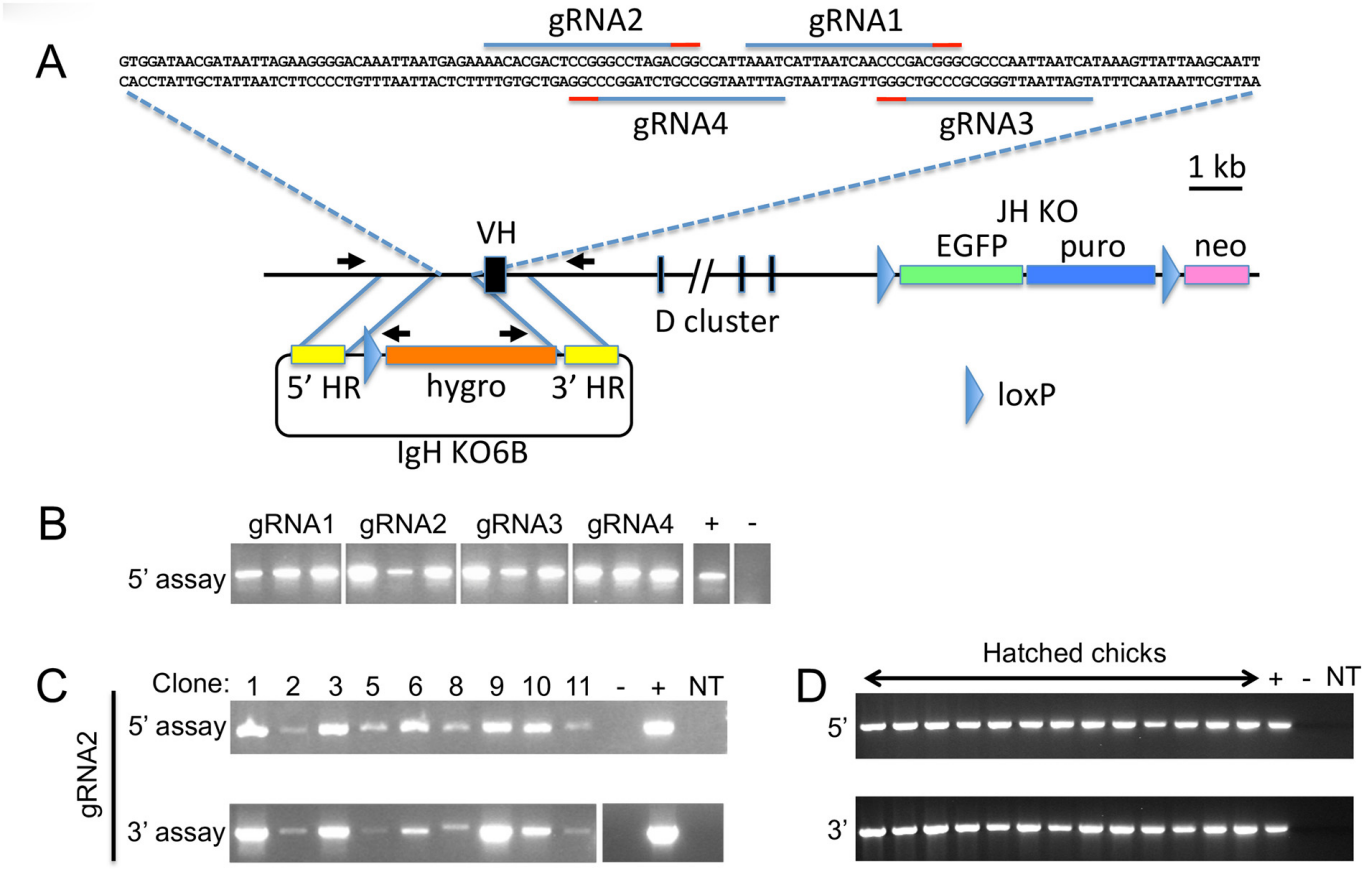
CRISPR-mediated targeting of IgH KO6B in PGCs. A. Detailed diagram of the IgH locus. The 122 bp sequence between the 5’ and 3’ homology regions in IgH KO6B, used to design the gRNAs, is shown at top. The locations of gRNAs 1–4 are indicated with blue lines above the sequence and the protospacer adjacent motifs (PAM) indicated with red lines. The repair vector IgH KO6B (below) contains 5’ and 3’ homology regions (HR) in yellow, a single loxP site (blue arrowhead), and a hygromycin selection cassette (orange). The locations of the primer binding sites for the 5’ and 3’ targeting assays are shown as black arrows. The downstream selectable markers in the JH-KO consist of floxed EGFP (green box) and puro gene (blue box), and a promoterless neo gene in opposite orientation (pink box). The loxP sites are blue arrowheads. B. The 5’ targeting assay performed on independent, non-clonal cell populations obtained from co-transfection of the four different gRNAs into 472–138 cells along with Cas9 and IgH KO6B. For each gRNA transfection, 3 hygromycin-resistant populations were analyzed. The positive control (+) was a DT40 cell line that contained a knockout of the functional V region [22] and the negative control (-) was the parental IgH KO6B plasmid. C. The 5’ and 3’ targeting assays performed on 9 independent clones obtained with gRNA2 (there were 12 clones, but clones 4, 7 and 12 grew more slowly and were not tested at this time). Variation in band intensity is likely to be from variation in the template gDNA amount, since the number of cells harvested was not normalized. The negative control (-) was genomic DNA from a JH-KO transgenic bird, and the positive control (+) was a pool of cells (G2) from the gRNA2 experiment in B. NT, no template control. D. The same 5’ and 3’ targeting assays performed on EGFP+ birds obtained from breeding cell line 1783–10 chimeras to wild type.

We chose to target the region upstream of the single immunoglobulin heavy chain variable region (VH) in the JH-KO cells to introduce a loxP site into the IgH locus. Four guide RNAs were designed to direct double-strand cutting of the genome by Cas9 at a site approximately 300bp upstream of the translation initiation site of the VH (Fig 2A) and each one was cloned separately into the GE6 vector with Cas9. The Cas9 cut sites are all approximately 50bp from the homology regions in the donor targeting vector, IgH KO6B. IgH KO6B was constructed with short homology regions of ~1kb flanking a hygromycin selection cassette (Fig 2A). Although the chicken IgH locus consists of only short stretches of sequence in the genome database, not organized into contigs, there was sufficient unique sequence available to design PCR primers and amplify these short homology regions. The homology regions were amplified from homozygous JH-KO genomic DNA and are thus isogenic for the allele that contains the JH-KO in the 472–138 cell line. The other allele in these cells is likely to be polymorphic since the bird used for deriving cell line 472–138 was outbred.

The 4 gRNAs to VH were separately co-transfected with circular IgH KO6B and stable transformants were selected with hygromycin. In the first set of transfections, we used 15μg of each plasmid (IgH KO6B and gRNA/Cas9) for 5 x 10^6^ cells, amounts of DNA that would normally yield approximately 1–10 colonies per 48-well plate when using linearized targeting vectors alone. However, when IgH KO6B was used in combination with gRNA/Cas9, the transfections were so efficient that every well for all 4 transfections contained multiple clones of hygromycin-resistant cells. Although these were not clonal populations, 3 wells from each transfection were harvested to test for targeting by IgH KO6B, and all contained the correct targeting event (Fig 2B). With gRNAs 1, 3 and 4, most wells had perhaps 4–5 colonies per well, suggesting that the targeting efficiency was, at worst, 20–25% (if there were only 1 positive clone out of 4–5). With gRNA2, most wells only had ~2–3 clones, suggesting a higher potential efficiency of ~33%, so for subsequent transfections we used gRNA2, and used lower amounts of the donor IgH KO6B to reduce the number of resistant colonies. Transfection with 2.5μg IgH KO6B + gRNA2/Cas9 yielded 12 colonies per 48-well plate, 9 of which were screened for targeting. All 9 clonal populations had the correct targeting (Fig 2C). Transfection with 5μg of IgH KO6B + gRNA2/Cas9 yielded >50 clones, while a control transfection with 5μg IgH KO6B without the gRNA/Cas9 yielded only 2 colonies, neither of which was targeted correctly (data not shown).

**Fig 3:**
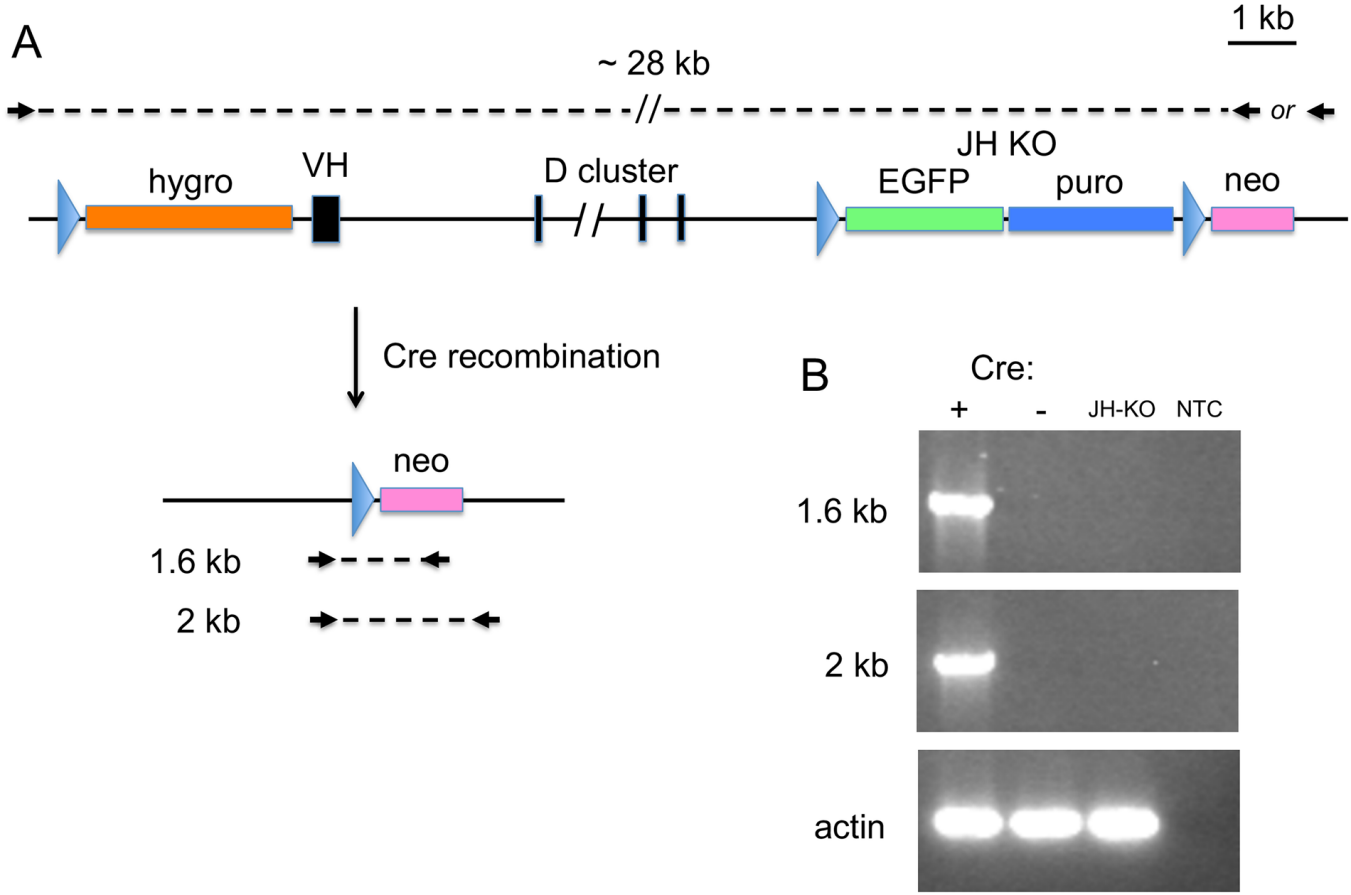
Cre recombination of CRISPR-targeted loxP site. A. Diagram of the targeted IgH locus before and after Cre recombination. A forward primer upstream of the CRISPR-targeted loxP site was used with two different reverse primers downstream of the loxP site in the JH-KO cassette. In the non-recombined allele, the forward and reverse primers are separated by about 28kb on the chromosome. After Cre recombination, a single loxP site and the promoterless neo gene remain, and the primers are either 1.6 or 2kb apart, which amplifies readily. B. PCR of recombined cells. Cre +: gDNA template from 1783–9 cells transfected with Cre; Cre -, parental 1783–9 cells; JH-KO, gDNA from a heterozygous JH-KO bird; NTC, no template control.

Targeting of the VH region was confirmed independently, taking advantage of the loxP site placed adjacent to the hygromycin gene in the targeted allele. The downstream JH-KO selectable markers are flanked by loxP sites (floxed) and should be on the same chromosome as the VH loxP site. If the targeting is correct the Cre recombination should excise the intervening DNA and leave behind a single loxP site and the promoterless neo gene (Fig 3A). Cells of the CRISPR-targeted clone 1783–9 were transiently transfected with a Cre-expression construct two times sequentially (to increase the percentage of cells with the excision) and cells were grown for four days after the second transfection to allow recombination. PCR primers lying outside of the loxP sites amplified the expected size products, whereas in unrecombined cells the primers are about 28kb apart, and no product was observed (Fig 3B), indicating that the CRISPR-targeted VH loxP site was in the correct position and orientation.

**Fig 4:**
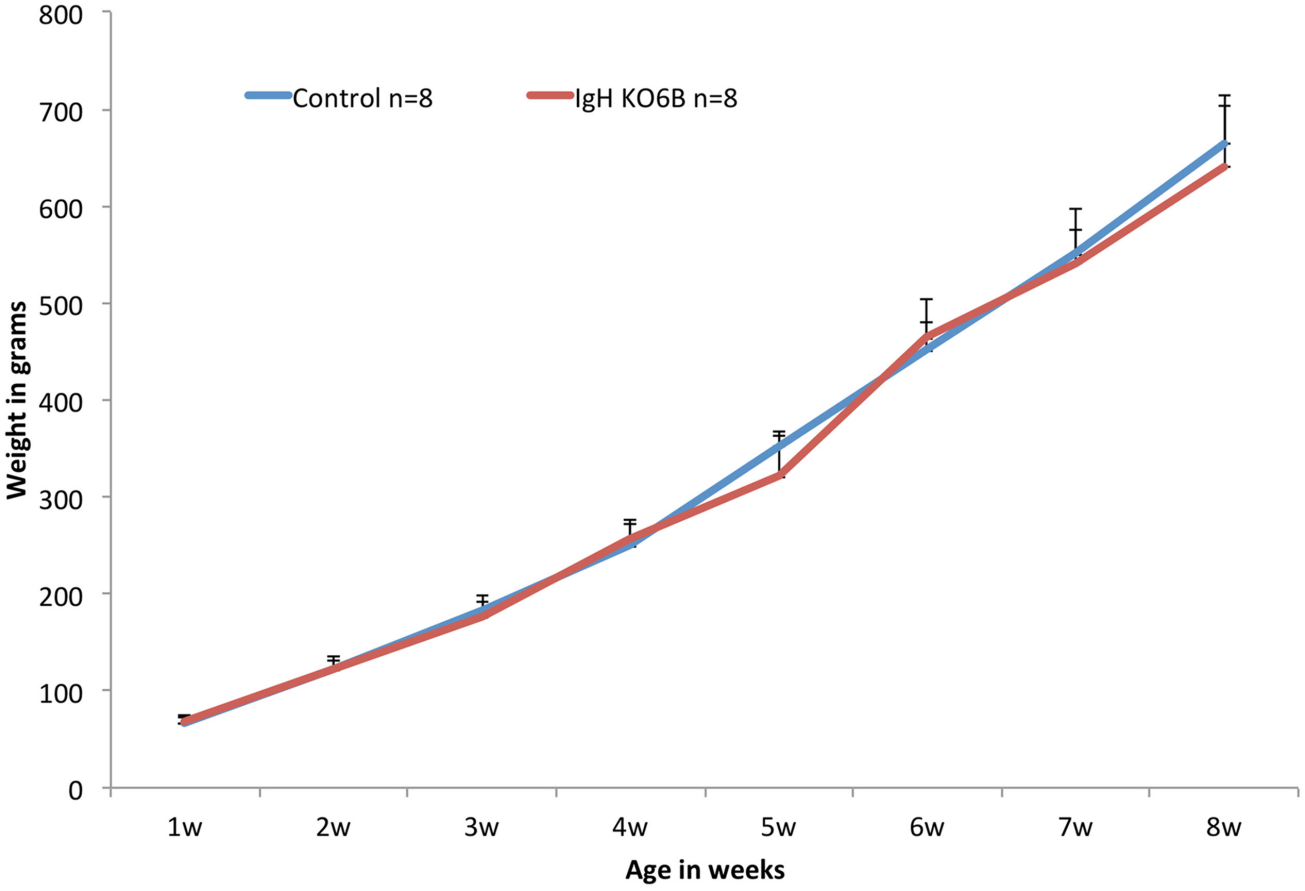
Growth curves of CRISPR progeny. Eight heterozygous IgH KO6B females and eight control females were weighed weekly for 8 weeks.

Five targeted cell lines (1783–1, 3, 6, 9 and 10) were injected into embryos to create germline chimeras [3]. Male chimeras were bred to wild type females and germline progeny screened by green fluorescence, using the EGFP transgene in the JH-KO selectable marker cassette. Four of the 5 cell lines transmitted germline progeny, to varying degrees (Table 1). One cell line, 1783–10, exhibited high rates of germline transmission, including one chimera with close to 100% transmission of the injected cells. Forty-six EGFP-positive progeny from transmission of cell line 1783–10 were hatched and typed for the CRISPR-targeted IgH KO6B. All of the EGFP-positive birds contained the IgH KO6B insertion (a subset is shown in Fig 2D), confirming that the PGC clones contained the correct stable integration. Heterozygous IgH KO6B birds hatched and grew normally (Fig 4).

**Table 1:** Germline transmission rates of chimeras made with CRISPR-modified PGCs.

Cell line	Chimera	# progeny screened	% germline transmission
1783–1	20649	29	0
1783–3	20622	305	8.6
1783–6	20626	19	0
	20638	175	16
	20643	35	0
1783–9	20455	37	0
	20457	25	0
	20492	27	0
	20499	40	5
	20642	149	15
1783–10	20619	130	96
	20623	34	12
	20646	154	36

Chimeras (bird IDs are listed) were germline tested by screening progeny for EGFP expression. % germline transmission was calculated as %EGFP^+^ progeny x 2, since EGFP was heterozygous in the PGCs injected.

## Discussion

The use of gene editing in bird species poses particular challenges based on the structure of the fertilized egg and early embryo. In other animals, it is possible to inject editing components (DNA, RNA or protein) directly into the zygote, but this would not be easily accomplished in birds. To access the germline, it might be possible to introduce CRISPR into embryo PGCs *in vivo* using a lentivirus or transposon, but the efficiency of transduction would need to be very high. In previous studies with TALENs [4], and in the current study using CRISPR/Cas9, cultured PGCs were genome-edited *in vitro* and re-introduced into the germline to produce progeny efficiently. The germline transmission frequencies observed in this study were variable, but consistent with the frequencies observed with other cell lines in our lab [3]. It is, therefore, important to screen several cell lines carrying a particular genetic modification for germline transmission. Transmission of more than one cell line may also be advantageous in case of off-target effects, to be able to select founders with the fewest off-target mutations. The normal growth rate of the heterozygous birds indicates that no dominant mutations affecting overall health were introduced by off-target effects of CRISPR. However, we did not test for off-target mutations and thus cannot rule out mutations that could lead to more specific phenotypes in these birds or to effects in future generations. Further study of off-target mutations at selected sites or in the whole genome will be necessary to determine the extent of these mutations. Any off-target effects not genetically linked to the targeted locus would be eliminated by Mendelian segregation.

The sequences surrounding the VH region are not well characterized in the chicken [23], limiting the ability to design long homology regions that would be required for a traditional gene targeting approach. In particular, the region just downstream of the VH contains multiple copies of an 80bp repeat which would be difficult to clone and potentially problematic to use in a homology region. The use of a site-specific endonuclease in combination with a repair vector enables the use of 2kb of total homology, which should be easily obtainable as a unique sequence for most loci of interest. The high frequency of targeting in chicken PGCs (100% in this study with CRISPR/Cas9) should allow even shorter regions of homology to be used successfully. HDR is often performed with oligonucleotides of ~100nt [24].

We pursued the HDR strategy with a drug-selection cassette in order to introduce a loxP site in the IgH locus. This approach has the added advantage that clonal populations of edited cells are produced, removing the mosaicism inherent in non-selected editing experiments such as direct injection of CRISPR/Cas9, and increasing the chances of obtaining germline transmission of the desired genetic changes. Any selectable markers in the offspring can be removed immediately by breeding the chimera to hens carrying Cre recombinase [25]. It may be possible to transmit through the germline mutations produced in bulk, non-selected populations of edited PGCs, but it would require screening many progeny if only a small percentage of the injected cells carry the mutations at the desired locus. Alternatively, PGCs could be single cell cloned by limiting dilution without drug selection to produce pure populations of modified cells, but the effect of prolonged culture, in less than desirable conditions, on germline transmission is unknown. The above described HDR approach is efficient, supports high rates of germline transmission, allows any gene edit to be designed precisely, and enables insertional mutagenesis and knock-ins.

## Materials and Methods

### Animals

Commercial White Leghorn chickens were obtained from Hendrix ISA, and Minnesota Marker Line chickens were from the Pacific Agri-Food Research Centre, Agassiz, British Columbia, Canada. Animal experiments were done in strict accordance to IACUC approved protocols and under supervision of the Crystal Bioscience IACUC committee. No animals became ill or died during the course of the experiments.

### Guide RNA design

A 159bp region upstream of the chicken functional heavy chain V was analyzed on the MIT server (http://crispr.mit.edu) for guide RNA design. Four guide RNAs were selected, synthesized and cloned separately into the GE6 vector containing the wild type Cas9 nuclease (Horizon): gRNA1, AAATCATTAATCAACCCGAC; gRNA2, AACACGACTCCGGGCCTAGA; gRNA3, TGATTAATTGGGCGCCCGTC; gRNA4, ATTTAATGGCCGTCTAGGCC. These gRNAs had few predicted off-target sites, none of which were in known coding sequences. A control construct containing gRNA5 from Shalem et al. [21] specific for EGFP was also made (AAGTTCGAGGGCGACACCC).

### Targeting vector IgH KO6B design

Homology regions of 1133bp and 1011bp were PCR amplified and cloned from a homozygous knockout chicken that carried the original JH-KO [8]. The 5’ HR was amplified with primers 5’-GCCCCTAATAAGTGGTTTAATTATG-3’ and 5’-TCTGCGCTGAGTTCTTTGAT-3’; the 3’ HR was amplified with primers 5’-AAGTCGAGGCTGACGAGAAA-3’ and 5’-CTTTTCCCCACCAAATTTCA-3’. Homozygous DNA ensured that the homology regions would be isogenic to the allele carrying the JH-KO in the cells used for targeting, 472–138, which are heterozygous for the JH-KO. The two alleles are likely to be polymorphic since the chickens used to derive these PGCs are outbred. In the IgH locus, the homology regions are separated by a stretch of 122 bp, which contains the sequences targeted by the gRNAs, ensuring that the gRNAs will target only the genome and not the targeting vector itself when they are co-transfected into the cells. The homology regions flank a β-globin HS4-insulated hygromycin-resistance gene for selection in PGCs and a loxP site designed to recombine with the downstream loxP sites in the JH-KO selectable marker cassette.

### Cells used for targeting

PGC line 472–138 contains a previously targeted heavy chain locus in which the JH region was replaced with a floxed selectable marker cassette. The cell line was derived by breeding a germline chimeric male, which had been injected with JH-KO PGCs, to a wild type hen and culturing cells from the germinal crescent of a Stage 4–8 (Hamburger and Hamilton) EGFP-positive embryo. PGCs were cultured as described [6]. Briefly, PGCs were grown in KO-DMEM (Life Technologies), of which 40% was preconditioned on buffalo rat liver cells (BRL, ATCC), and supplemented with 7.5% fetal bovine serum (Hyclone), 2.5% irradiated chicken serum, 1X non-essential amino acids, 2mM glutamine, 1mM sodium pyruvate, 0.1mM β-mercaptoethanol (all from Life Technologies), 4ng/ml recombinant human fibroblast growth factor, 6ng/ml recombinant mouse stem cell factor (both from R&D Systems) and grown on an irradiated feeder layer of BRL cells. The cells were passaged 3 times per week onto fresh feeder layers.

### Transfection and injection

To test for inactivation of the EGFP locus, 15μg of either EGFP-specific gRNA5/Cas9 or Cas9 alone were added to 3 x 10^6^ cells and brought to a volume of 100μl with V-buffer (Lonza, Walkersville). The cell suspension was transferred to a 2mm cuvette and subjected to 8 square wave pulses of 350 volts/100μsec (BTX 830 electroporator). After electroporation the cells were resuspended in culture medium and cultured for 9 days to allow the remaining EGFP in the cells to dilute out. Cells were analyzed for loss of green fluorescence using the Attune flow cytometer (Life Technologies). For stable transfectants targeting the IgH locus, 15μg of circular gRNA1, 2, 3 or 4/Cas9 and 2.5, 5 or 15μg circular IgH KO6B were added to 5x10^6^ cells and transfected as described above, then plated with hygromycin-resistant irradiated BRLs and seeded in a 48-well plate at a density of 10^5^ cells per well. A control transfection with 5μg IgH KO6B without gRNA/Cas9 was also done. After 3 days, 40μg/ml hygromycin was added to select for cells with a stable integration of IgH KO6B. After stable clones were identified, the cells were expanded and confirmed for the IgH KO6B integration by PCR. Confirmed clones were injected into recipient chicken embryos at Stage 14–16 (H&H). The injected embryos were transferred to surrogate shells and incubated until hatch at 37°C. The sex of the chicks was determined after hatch by PCR for the W-chromosome.

### Screening for targeting by IgH KO6B

Hygromycin-resistant clones were analyzed by PCR for targeting by IgH KO6B. For the 5’ assay, the forward primer was chVH-F5: 5’-TGGTTTGGTTGATGGAAGAATGTA-3’ and the reverse primer was HA-R: 5’-ATACGATGTTCCAGATTACGCTT-3’. For the 3’ assay, the forward primer was KO 6B-F: 5’-GCTGAACTAGAATGCATCAAGC-3’ and reverse primer chVH-R33: 5’-ACAAACCTTTGCCGCATCCA-3’.

### Cre recombination of inserted loxP site in the IgH locus

3x10^6^ Cells from line 1783–9, carrying the CRISPR-targeted loxP site and JH-KO loxP sites, were transiently transfected with 20μg of a β-actin-Cre expression construct as described above, and cultured for 10 days. The Cre transfection was then repeated to increase the percentage of cells with the excision, and four days later the cells were harvested for PCR analysis of Cre/lox recombination between the two outermost loxP sites. Two PCR assays were performed: both used a 5’ primer in the upstream VH flanking region (chVH-F3aB: 5’-GATGGGGGGTGGCAATGGAATGAT-3’). The 3’ primer was located either in the neo gene in the JH-KO (neo-F1: 5’-AGCTGTGCTCGACGTTGTCACT-3’) generating a 1.6kb amplicon, or in the IgH locus downstream of the selectable markers (chJC-R45: 5’-GCCCAAAATGGCCCCAAAAC-3’), generating a 2kb amplicon.

### Germline transmission of CRISPR-treated cells

Male chimeras were grown to sexual maturity and bred to wild type hens. Hatched chicks were evaluated for the expression of EGFP, and the germline progeny were confirmed by PCR to carry the targeted IgH KO6B using the assays described above.
